# Using Social Network Sentiment Analysis and Genetic Algorithm to Improve the Stock Prediction Accuracy of the Deep Learning-Based Approach

**DOI:** 10.1007/s44196-023-00276-9

**Published:** 2023-05-29

**Authors:** Jia-Yen Huang, Chun-Liang Tung, Wei-Zhen Lin

**Affiliations:** Department of Information Management, National Chin-Yi University of Technology, No.57, Sec. 2, Zhongshan Rd., Taiping District, Taichung City, 41170 Taiwan, ROC

**Keywords:** Stock prediction accuracy, Genetic algorithm, Social media sentiment, COVID-19 pandemic, Deep learning, Taguchi method

## Abstract

Traditionally, most investment tools used to predict stocks are based on quantitative variables, such as finance and capital flow. With the widespread impact of the Internet, investors and investment institutions designing investment strategies are also referring to online comments and discussions. However, multiple information sources, along with uncertainties accompanying international political and economic events and the recent pandemic, have left investors concerned about information interpretation approaches that could aid investment decision-making. To this end, this study proposes a method that combines social media sentiment, genetic algorithm (GA), and deep learning to predict changes in stock prices. First, it employs a hybrid genetic algorithm (HGA) combined with machine learning to identify chip-based indicators closely related to fluctuations in stock prices and then uses them as input for long short-term memory (LSTM) to establish a prediction model. Next, this study proposes five sentiment variables to analyze PTT social media on TSMC’s stock price and performs a grey relational analysis (GRA) to identify the sentiment variables most closely related to stock price fluctuations. The sentiment variables are then combined with the selected chip-based indicators as input to build the LSTM prediction model. To improve the efficiency of the LSTM analysis, this study applies the Taguchi method to optimize the hyper-parameters. The results show that the proposed method of using HGA-screened chip-based variables and social media sentiment variables as input to establish an LSTM prediction model can effectively improve the prediction accuracy of stock price fluctuations.

## Introduction

Taiwan’s stock market continues to grow in scale, with the number of listed companies increasing from 698 to 954 between 2007 and 2021. Investors must, thus, make more rational investment decisions to efficiently select stocks worthy of investment from numerous targets. Early research has mainly adopted random walk theory (RWT), which claims that stock prices follow a random walk model and cannot exceed 50% accuracy prediction [21]. Chen [10], however, pointed out that RWT is not suitable for the Taiwan Weighted Stock Index, and investors can predict the direction of future stock price movement from past stock price data. Schumaker et al. [39] stated that, using statistical and mathematical methods, value investment targets can be identified from a large amount of historical data to obtain stable, sustained, and above-average investment returns.

Data for various quantitative technical indicators, such as trading volume, earnings per share, and price–earnings ratio, are openly available to the public. These indicators are not only the basis for investors to gage the movement direction of stock prices but also are often used by academicians to study stock price forecasts [52]. Studies have applied various machine learning tools to analyze changes in the stock market using technical indicator data. However, an excess of indicators is not necessarily conducive to prediction. Given that GA is a powerful machine learning tool for global searches, this study uses GA to screen important indicators leading to stock price fluctuations and further applies the recently emerged deep learning (DL) to predict stock prices.

Major international political and economic events, from the 2008 financial crisis to the COVID-19 pandemic in recent years, as well as the US–China confrontation and the Ukrainian–Russian war, have affected stock price volatility, rendering forecasting increasingly difficult. News related to such events or company operations often influences investors’ stock investment decisions. Several relevant research has shown that in addition to the quantitative variables of finance-related indicators, news information affects investors’ investment decisions, which in turn affects the change in stock prices [6, 7, 14, 43]. In addition to information from traditional media, investors are referring to opinions on social media or sharing their views on various online platforms. Thus, in addition to finance-related indicators, investor sentiment is an important variable when estimating stock prices.

In recent years, information technologies, such as text mining and web crawlers, have enabled social media comments to be automatically and objectively collected and processed on a large scale. Many scholars have converted news data from social media into quantitative sentiment information to predict stock price changes using machine learning [45]. Using the Chinese language for a sentiment analysis can be difficult, given the need for text segmentation. Moreover, the sentiment dictionary resources are incomplete and difficult to construct [31]. Therefore, this study focuses on a sophisticated sentiment analysis technique that converts text from social media into predictive variables to help improve the forecasting accuracy for stock prices.

Stock price forecasting is a challenging task, given the volatile and non-linear nature of financial stock markets. To tackle this complex problem, this study proposes a complete and innovative forecasting framework comprising feature selection (FS) that combines GA and machine learning tools. In addition, it uses the Taguchi method to find the optimum configuration of DL network architecture, identify sentiment variables most strongly correlated with stock fluctuations, integrate the technical indicators and sentiment variables in the DL model, and assess the impact of COVID-19 on prediction accuracy.

The remainder of this paper is organized as follows. Section 2 reviews machine learning prediction models that are based on technical indicators related to the stock market and the use of social media mining to improve prediction models. This section also illustrates the importance of feature selection in forecast accuracy and reviews the literature on predictive modeling using the results of feature selection. Section 3 details opinion mining of social media and the proposed sentiment variables. It then develops the method of applying HGA to screen chip-based indicators that cause fluctuations in Taiwan Semiconductor Manufacturing Company’s (TSMC) stock prices. Section 4 uses the selected indicators to determine the highest prediction accuracy using LSTM. This section performs a grey relational analysis (GRA) to select the sentiment variables most closely associated with stock price volatility and then adds the variables to the LSTM model to evaluate their effect on improving prediction accuracy. Section 5 presents conclusions and recommendations for future research.

## Literature Review

This section reviews research that applied machine learning techniques, features selection, and social media mining to predict stock prices.

### Machine Learning Prediction Model Based on Technical Indicators Related to Stock Markets

Early research primarily used statistics and machine learning techniques to forecast stock prices. However, the forecasting performance of these statistical methods is not satisfactory. Autoregressive integrated moving average model (ARIMA), for example, often requires more historical data to satisfy the statistical assumptions of normality. Empirical results show that machine learning techniques have superior predictive power over statistical models because they can identify hidden relationships between factors affecting stock markets and capture complex patterns in data without prior knowledge from input data [2].

There are various tools that can be employed in machine learning to predict stock market changes on the basis of technical indicator data, and each tool has its own advantages. Common tools include support vector machine (SVM), logistic regression (LR), artificial neural network (ANN), and deep learning [35]. Ballings et al. [5] compared various classifiers used to predict the fluctuation in stock prices and concluded that the use of tools for forecasting has positive implications for investments. LR, which classifies data by probability value, has the advantages of strong adaptability, robustness, and good model interpretability. Huang and Liu [21] selected more than 50 technical indexes, used LR to screen variables, and accordingly, established a predictive stock price model. SVM has high prediction accuracy and reports good performance in solving problems, such as small sample sizes, nonlinearity, and high dimensionality. Endri et al. [15] used SVM to develop an early warning system to predict the delisting of Islamic stocks (ISSI). Chen and Hao [12] proposed a hybrid framework using feature-weighted SVM and K-nearest neighbor (KNN) to predict stock market indices.

The use of evolutionary computation, especially GA, is not uncommon in the financial literature [3]. GA searches for optimal global solutions by imitating the concept of survival of the fittest, and its application range continues to expand extensively [3]. Since the algorithm is a variant of adaptive probabilistic search technologies, its selection, crossover, and mutation operations are performed from a probability viewpoint, thereby increasing the flexibility of its search process. Chung and Shin [13] used GA to optimize the parameter settings of neural networks, and Sezer et al. [40] and Sezer et al. [41] applied GA to optimize the technical indicators of stock markets. However, these studies adopt the simplistic approach of basing buying and selling points on technical indicators for the relative strength index (RSI).

Given its excellent predictability in image classification and natural language processing, deep learning has attracted widespread attention and has been applied to stock prediction in recent research. Representative methods of deep learning include deep belief networks (DBN), convolution neural networks (CNN), recurrent neural networks (RNN), and LSTM. LSTM is an improved RNN, which is one of the most advanced deep learning algorithms. However, relevant studies are insufficient in the field of financial forecasting. Li and Tam [30] used SVM and LSTM to predict the performance of various stocks listed on the Shanghai Stock Exchange (SSE 50 Index). Their results showed that SVM is suitable when forecasting low volatility stocks, and LSTM has the best overall performance for stocks ranging between medium and high volatility. Zhao et al. [54] used the Random Forest (RAF) regression algorithm for feature screening, and proposed a hybrid model which combined an economic model with a deep learning model to improve the prediction accuracy of the option pricing model of CSI 300ETF.

Singh and Srivastava [43] argued that stock market forecasting is a chaotic, complex, volatile, and dynamic time series problem. Given the failure of existing ANN methods to provide encouraging results, they proposed that deep learning can improve the accuracy of stock market forecasts. Fischer and Krauss [16] used the LSTM network to predict the price movement direction of S&P 500 constituent stocks from 1992 to 2015 and showed that the LSTM network outperforms RAF, deep neural networks (DNN), LR, and other classifiers. Bao et al. [6] proposed a novel deep learning framework that incorporates methods, such as stacked autoencoders, wavelet transforms, and LSTMs, to predict stock prices.

Chung and Shin [13] pointed out that although LSTM is a powerful tool to solve time series and pattern recognition problems, they are subject to certain shortcomings. First, LSTMs cannot provide specific explanations for their prediction results. Second, like other neural network models, LSTMs have many parameters that must be tuned, such as the number of layers, neurons per layer, and the number of time lags. However, time and computational constraints make it impossible to examine all parameter spaces and find the optimal parameter set, and thus, the setting of these control parameters often depends on the researcher’s experience. To solve this problem, Kim and Shin [28] applied GA to assist both adaptive time delay neural networks (ATNN) and time delay neural networks (TDNN) to optimize time delay values and network architecture. Chung and Shin [13] used GA to optimize the temporal pattern of daily data for the Korean Stock Price Index (KOSPI) and detection-related architectural factors, such as time window size and the number of LSTM units in hidden layers. Chung and Shin [13] claimed that their LSTM network comprises two hidden layers and is characterized by deep architecture that can effectively express the non-linear and complex nature of stock markets. However, the optimal number of neurons tends to differ when the number of hidden layers varies.

### Features Selection

In recent years, numerous studies from a wide range of fields have applied FS to improve the accuracy and efficiency of predictive models [42]. This section briefly reviews research on GA-based features engineering in non-financial fields. Yang et al. [51] present a fault diagnosis approach comprising an FS method based on GA and then use selected features as input attributes for classification. Using a typical classification method, such as SVM and random forest (RF), they show that the optimized features space is superior to the original features space. Pei et al. [36] combine GA with RF cascaded for FS to expedite image classification and improve the accurate recognition rate. Khan et al. [27] apply GA to a texture-based feature descriptor to remove possible redundant features. Evaluating the proposed classification framework on a standard 7-class microstructural image dataset offers impressive outcomes, confirming its superiority over certain state-of-the-art methods.

FS can be applied in numerous ways to improve forecasting accuracy in finance-related fields. Yuan et al. [53], for example, use RF and SVM-recursive feature elimination (SVM-RFE) in their features engineering analysis to predict the Chinese stock market. Zhao et al. [54] use RF in their regression algorithm for FS and propose a hybrid model that combines an economic model with a DL model to improve the prediction accuracy of an option pricing model for CSI 300 ETF.

In addition to the above-mentioned machine learning methods, many studies have used GA with FS to improve prediction accuracy in finance-related fields. Abraham et al. [1] use stock prices recorded over the past 10 days and the historical data of four international stock indices (S&P 500, NIKKEI 225, CAC40, DAX) as the chromosomes for their features engineering with GA and establish a prediction model with RF. They select 15 stocks across three sectors (technology, finance, and health) for stock price forecasting and find that the most relevant indicator for stock price changes is S&P 500. This result is not surprising given that most of the 15 stocks are US-based companies. Moreover, the authors treat stock movement prediction as a binary classification problem. They classify stock trends as an uptrend if the change in daily return price is greater than 0.5% and a non-uptrend otherwise. While the data for their study were collected for more than 17 months, the four stock indices rose more and fell less during their analysis period, creating an imbalanced dataset.

GA is one of the most widely used meta-heuristic and evolutionary algorithms in various domains, such as stock trend prediction and determination of optimal model configuration for DL. Sharma et al. [42] propose a method with ANN and GA for stock market forecasting. Their study uses historical data for DOW 30 and NASDAQ 100 and reveals that the accuracy of the hybrid model is greater than that of a single ANN technique. Considering the significant dependence of LSTM performance on architectural design, Suddle et al. [44] develop GA-based algorithms to automatically optimize the LSTM architecture for a sentiment analysis. Oyedele et al. [33] developed a GA-tuned framework to obtain a generalized prediction for the daily closing prices of cryptocurrency.

The literature on stock market forecasting commonly adopts feature selection algorithms, such as principal component analysis (PCA), stepwise regression analysis (SRS), and information gain and decision tree (DT). However, these feature selection algorithms are unable to determine the direct influence of stock features on stock price [4]. Researchers have also adopted other feature selection methods. Peng et al. [37], for example, discuss feature selection in the context of DNN models to predict stock price direction. They apply three feature selection methods to reduce the feature dimension from a set of 124 technical analysis indicators, namely the sequential forward floating selection (SFFS) algorithm, tournament screening (TS) algorithm, and least absolute shrinkage and selection operator (LASSO). Among these, TS is analogous to a genetic algorithm. Given the success of GA in solving complex optimization problems, researchers from various fields, including image classification, medical gene identification, and visual human action recognition have used GA for feature selection [38]. However, few studies discuss the application of GA to feature selection in stock market prediction.

The work of Huang and Liu [21] is the most analogous to our study. Focusing on stock price forecasting, their research uses chip-based indicators as predictive variables and conducts feature selection. They select more than 50 technical indexes, use LR to screen variables, and accordingly, establish a predictive stock price model. However, it is difficult to describe the moving tendency of stock prices given their non-linear, non-stationary, and noisy characteristics [4]. In view of the nonlinearity in stock data, a model developed using a traditional or single intelligent technique may not accurately forecast results [42]. LR cannot solve nonlinear problems and is sensitive to multi-collinear data, thus, many studies have stated that it is inappropriate to use LR for feature selection. Wu et al. (2022) [50], for example, highlight the difficulty of using LR to screen features and that the quality of feature engineering cannot be guaranteed. Therefore, they propose a gradient-boosting decision tree algorithm to improve the quality of feature engineering.

The literature review presented thus far highlights the positive impact of GA in features engineering across fields. Thus, this study adopts the feature selection method with GA. Another important step in performing a GA analysis is chromosome setting. Each study uses different variables to set chromosomes, and this autonomy allows for research creativity. This study is the first to use chips-based indices as the genes that make up the chromosome.

### Integration of Social Media Mining and Stock Price Prediction

In addition to chip-based indicators, stock markets are affected by other uncertainties, such as opinions shared through news and social media. News and social media have become valuable resources when mining public sentiment using text sentiment analysis technology. Predicting stock price fluctuations by mining news texts is an emerging field of data mining research. Several studies have abandoned the traditional method of predicting stock prices on the basis of technical variables and simply explored the relationship between news sentiment and stock prices or combined technical variables and news sentiment to predict stock prices [6]. Nti et al. [32] presented a detailed review of research combining technical variables and social media sentiment to predict stock market trends.

Schumaker et al. [39] developed a stock price prediction engine to analyze financial news sentiment. The experimental results showed that the investment performance of this system is better than that of the market average. Peng et al. [37] applied features extracted from historical price data and financial news to DNNs to predict stock movements and showed that financial news could significantly improve forecasting accuracy. Cakra and Trisedya [8] examined the sentiment of Twitter posts to predict the direction of Indonesian stock market volatility. Pagolu et al. [34] studied the correlation between the emotional state of Twitter users and the price of the Dow Jones Industrial Average (DJIA) and found a strong correlation between the two.

Vargas et al. [46] applied deep learning to predict daily stock price movements on the basis of financial news headlines and technical indicators. In addition to comparing two sets of technical indicators, their study compared a financial news hybrid model composed of CNN, an LSTM hybrid model for technical indicators, and an I-RNN model comprising technical indicators. Their results showed that CNN is better than RNN in extracting semantics from text, while RNN performs better in capturing contextual information and in simulating complex temporal characteristics. In addition, financial news plays a major role in obtaining stable results, while the two sets of technical indicators have little effect on forecast accuracy. Wu et al. [50] combined stock forum posts, financial news, technical indicators and stock historical transaction data as the feature set of stock price prediction and adopt the LSTM to predict the China Shanghai A-share market. Since only three technical indicators were considered in their study, including the stochastic oscillator index, William index and relative strength index, their accuracy is still far from sufficient [29].

Since information on stock purchases and sales in Taiwan is publicly available, it is not uncommon for academicians to use technical variables to make stock selection decisions in the context of the Taiwan Stock Exchange. Huang et al. [24], for example, used a wrapper approach to select the best subset of features from an original feature set containing 23 technical indicators and then applied a voting scheme combining different classification algorithms to predict the trend of the Korean and Taiwanese stock markets. Wei et al. [47] constructed an integrated news sentiment index (ANSI) on the basis of Chinese financial news related to all companies listed on the Taiwan Stock Exchange. A rise in the index is accompanied by an increased trade value and a reduction in investor fear, as indicated by the Taiwan volatility index (TVIX). Their findings proved that sentiment levels reflected in news reports could be effectively used as a reference for investment decisions. Chang et al. [9] examined 50 Taiwanese constituent stocks using a vector auto-regression model and showed that Taiwan’s large-capitalization stocks are most affected by corporate and foreign investments. Wu et al. [49] showed that news variables provide information that is useful in predicting Taiwan stock market returns, and the prediction accuracy is higher when the stock market is booming. Huang and Liu [21] used binary LR to establish a forecasting model for three price-level changes (0%, 0.5%, and 1.0% and above) for the stocks of Taiwan Hon-Hai Precision Industry Co., Ltd. (HHPIC). However, their analysis results were not statistically significant owing to the relatively small data. Huang and Liu [21] integrated chip-based indicators and sentiment variables with their LR forecasting model and claimed that sentiment variables could be used to improve forecast accuracy. However, they did not evaluate the impact of the selected variables on forecast accuracy, and the selected variables changed when predicting the percentage of price fluctuation for different stocks.

In sum, the accuracy of stock prediction is closely related to the selection of predictor variables. Thus, this study proposes the use of GA to screen chip-based indicators and combines the results with sentiment variables as input features for deep learning to build a prediction model. Since the collected data span the period before and after the pandemic, this study also compares changes in important chip-based indicators and social media sentiment before and after the pandemic.

## Methodology

### Research Framework

**Fig. 1:**
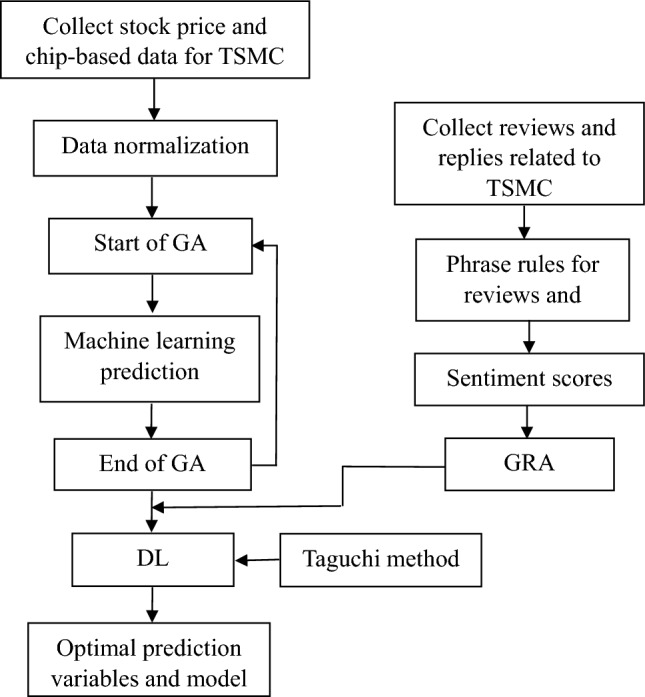
Research framework

This research references two types of data: chip-based indicators and social media data. The first is obtained from the Taiwan Stock Market Observation Post System for TSMC, and the second is reviews and corresponding replies posted about TSMC on PTT’s bulletin board. Both data were collected between January 1, 2019, and April 31, 2021. This study performed a pre-process and word segmentation on PTT’s corpus and then extracted opinion words to establish quantitative variables for social media sentiment. It then applied a hybrid GA to screen the chip-based variables related to price fluctuations for TSMC’s stock and used LSTM to establish the prediction model. Figure 1 illustrates the research framework.

### Data for Chip-Based Indications

Data collected for TSMC’s 25 chip-based indicators can be divided into four categories: transaction information, buy/sell information for three major institutional investors, margin /stock loan, and day trading information (see Table 1).

**Table 1:** List of collected chip-based indicators

Categories	Indicators			
Transaction information	Turnover in value (TIV)	Trading volume (TV)	Number of shares traded (NST)	Price fluctuation limit (PFL)
Buy/sell of three major institutional investors	Foreign/Chinese investors buy (FCIB)	Securities investment trust buy (SITB)	Dealer buy (DB)	Net buy/sell of three institutional investors (NBSTII)
	Foreign/Chinese investors sell (FCIS)	Securities investment trust sell (SITS)	Dealer sell (DS)	
Margin loan/ Stock loan	Margin loan buy (MLB)	Stock loan buy (SLB)	Margin loan cash pay (MLCP)	Margin loan balance (MLB)
	Margin loan sell (MLS)	Stock loan sell (SLS)	Stock loan cash pay (SLCP)	Stock loan balance (SLB)
	Margin balance long (MBL)	Margin balance short (MBS)	Day offset of margin purchasing and short selling (DOMPSL)	
Day trading information	Day trading turnover (DTT)	Day trading volume (DTV)	Day trading Number (DTN)	

Referencing Hong et al. [17], this study sets February 21, 2020, as the cut-off date for the occurrence of COVID-19. Therefore, the period before the pandemic is from January 1, 2019, to February 20, 2020, and that after the pandemic is between February 21, 2020, and April 29, 2021. The overall period includes 561 trading days, excluding the dates when the stock market was closed. The period after the pandemic has 290 trading days.

### Opinion Mining of Social Media

Globally well-known social media platforms, such as Facebook, YouTube, Line, and Instagram, have their own main functions and features that attract users. PTT is built on the resources of Taiwan’s academic network and serves as an instant online discussion platform. It is one of the most influential and widely used online forums in Taiwan [11], with good features for instant interactions and rapid dissemination speed. The platform has 1.5 million registered users who contribute more than 20,000 reviews and 500,000 replies per day. The most frequent users belong to the age group of 20–45 years, and most of them are employed and are financially able.

Articles on PTT are composed of two parts: reviews and replies to the reviews. The author of an article comments on a specific subject in the form of a review, and users post responses to the review as replies. Both parts use different grammatical structures given the purpose and professional quality of users. Most review authors have strong professional literacy in the stock market and express their views on current affairs with longer and more rigorous phrases and professional words. Replies, on the other hand, reflect users’ feelings in response to a review. The content is relatively brief and casual, and the grammatical structure considerably differs from that of reviews. To analyze sentiments on social media, this study referred to Huang and Liu’s [21] method to establish phrase rules for reviews and to Huang and Lu’s [20] approach to determine phrase rules for replies.

This study collected TSMC-related information from 2,177 reviews and about 30,000 replies on PTT. Since the occurrence of degree words in replies is relatively low, this study set degree words to three levels (i.e., over, very, and extreme) and assigned the levels 1, 2, and 3 points, respectively. When negative words, such as “no” and “does not have”, appeared in a sentence, the positive and negative meanings of the sentence were likely to be reversed. If negative words were used before or after positive opinion words, then the sentence was classified as representing negative emotions. However, if negative words appeared before or after negative opinion words, the sentence was classified under expressions of positive emotions.

Based on the assumption that all replies reflect the same emotional direction as the review, Huang and Liu [21] estimated the sentiment score as the sum of the sentiment scores multiplied by the number of replies. However, while the replies were in response to the reviews, the polarity of emotions may not be in the same direction. Therefore, it is worth re-evaluating whether it is appropriate to consider the number of replies as a weight in calculating sentiment scores. To select suitable sentiment scores to enhance prediction accuracy, this study proposed five sentiment variables (see Table 2 for an example).

1. Positive sentiment score (PS): The variable assumes that the direction of TSMC’s stock movement is associated with positive sentiments on PTT, and its value is the sum of positive scores for reviews and replies on that day. As shown in Table 2, this variable scores 17 points on the basis of all reviews and replies that satisfy the phrase rules on that day.
2. Negative sentiment score (NS): This variable assumes that the direction of TSMC’s stock movement is related to negative sentiments on PTT, and its value is the sum of negative scores for reviews and replies on that day. The variable reports a score of − 15 points as per all reviews and replies that meet the phrase rules on that day (Table 2).
3. Sum of positive and negative scores (PNS): This variable summarizes whether the overall reaction of PTT users on a given day is positive or negative toward TSMC’s stock price. Its value is the sum of the positive and negative sentiment scores on that day. The score for this indicator on that day is 2 points (Table 2).
4. Huang and Liu sentiment score (HL): This variable, proposed by Huang and Liu [21], is based on the sentiment of a review, and the number of corresponding replies is used as the weight. The variable does not quantify reply sentiment. For instance, if the review sentiment score is 10 points and the number of replies is 30, the total sentiment score for the article is 300. The sentiment score for this variable is the sum of sentiment scores for all articles on a given day.
5. Jing sentiment score (JS): This variable refers to Jing et al.’s (2021) [26]scoring method, which records the number of positive and negative reviews on a given day and uses the total number of reviews on the day as the denominator to calculate the sentiment score of the day. It is estimated as the total number of positive reviews minus the total number of negative reviews divided by the total number of review articles on the day. This variable does not account for sentiment and the number of replies.

**Table 2:** Example of PTT’s sentiment score calculation for TSMC

	Number of reviews and replies that match phrase rules		Scores after considering degree words	
	Positive number	Negative number	Positive score	Negative score
Degree (score)				
Over (1)	3	4	3	− 4
Very (2)	4	1	8	− 2
Extreme (3)	2	3	6	− 9
PS score	17			
NS score	− 15			
PNS score	2			

Among the above-mentioned sentiment variables, this study used GRA to identify variables most related to fluctuations in TSMC’s stock price. Accordingly, it incorporated the variables into the forecast model to evaluate their effect on forecast accuracy. One of the major advantages of GRA is its ability to identify major correlations among factors of a system [19]. The steps for GRA are as follows:

1. Definition of reference sequence and comparability sequences: In this study, the reference sequence, *Y* = (*y*_1_,*y*_*2*_,…,*y*_*n*_), is the daily rise or fall records for TSMC’s stock price, and the comparability sequences, *X*_*i*_ = (*x*_*i,*1_,*x*_*i,2*_,…,*x*_*i,n*_), is the daily scores for the sentiment variables *i*, where *i* = 1,2,…,5, represents the five above-mentioned sentiment variables.
2. Normalization of comparability sequences: Using Eq. (1), *x*_*i,j*_ was converted into a number between 0 and 1, where *x*_*max i*_ and *x*_*min i*_ denote the maximum and minimum value of each sentiment variable.
  1 $$\overline{x}_{i,j} = \frac{{x_{i,j} - x_{\min i} }}{{x_{\max i} - x_{\min i} }}.$$
3. Calculation of grey relational coefficient $\varsigma_{ij}$ for each sentiment variable:
  2 $$\varsigma_{ij} = \frac{{\Delta_{\min } + \xi \times \Delta_{\max } }}{{\Delta_{ij} + \xi \times \Delta_{\max } }},$$

where, Δ_*ij*_ is the deviation sequence of the reference sequence (*Y*) and comparability sequence (*X*_*i*_), that is, Δ_*ij*_ =||*y*_*j*_-$\overline{x}_{i,j}$||, where *i* stands for sentiment variable and *j* denotes date. The distinguishing coefficient $\xi$ is set at 0.3. Δ_max_ is the largest value for Δ_*ij*_ and the Δ_min_ is the smallest value for Δ_*ij*_.

(4) Computation of grey relational grade: Grey relational grade Γ_*i*_ of each sentiment variable was estimated by averaging the grey relational coefficient corresponding to each sentiment variable:
  3 $$\Gamma_{i} = \frac{1}{n}\sum\limits_{j}^{n} {\varsigma_{ij} } ,i = {1},{ 2}, \ldots ,{5}.$$

### Hybrid Genetic Algorithm

This study focused on predicting if the increase in TSMC’s stock price is greater than 0%, which is a binary classification problem. Thus, it selected common classifiers in machine learning to establish prediction models, including decision tree (DT), LR, and SVM. The study incorporated GA with machine learning tools to find the optimal combination of chip-based indicators, and thus, it is called a hybrid genetic algorithm.

In the present HGA process, the initial population of chromosomes is randomly generated bit by bit. If a gene corresponding to a certain chip-based indicator is set to 0, the variable is not included in the classifier analysis. On the other hand, if it is set to 1, the variable is included. This selection mechanism simulates the evolutionary process; that is, the best chromosomes have more copies in the next generation, and the worst ones perish. The priority order for each chromosome during the evolution is based on its fitness function value, which is set to be the prediction accuracy of machine learning. Referencing Huang and Yao’s [25] elitism strategy, this study adopted the roulette wheel mechanism to select chromosomes for reproduction in the HGA. It directly copied 10% of the chromosomes with the best fitness values to the next generation. The step-by-step procedure for the HGA is as follows:

Step 1. Input the dataset and GA parameters with a chromosome length of 25 (i.e., the number of chip-based indicators); a crossover rate of 0.8; and a mutation rate of 0.003846, which is obtained following Huang and Wu’s [23] suggestion of 1/(chromosome length + 1). Set *gen* = 1 and *Rec.dat* = ∅.

Step 2. Randomly generate binary strings for the chromosomes in the initial population whose number is set to 100 groups.

Step 3. Simulate the evolutionary process by applying selection and reproduction, followed by crossover and mutation to the current population to generate a new population in the next generation.

Step 4. Check if each set of chromosomes exists in *Rec.dat*. If all the sets of chromosomes are recorded in *Rec.dat*, proceed to Step 5. Chromosomes that are not stored in *Rec.dat* are used by machine learning classifiers to build prediction models. Each chromosome is modeled on a ten-fold validation, and its prediction accuracy is averaged as the fitness function value.

Step 5. If the termination condition is satisfied, stop the evolutionary process and select the solution with the largest objective value in *Rec.dat* as the best solution. Otherwise, set *gen* = *gen* + 1 and go back to Step 3. When the evolutionary generation reaches 300, or the best solution has not improved in the last 50 generations, the GA operation is terminated.

Prediction accuracy (ACC) in Eq. (4) is based on the classification table of prediction models (Table 2) and is used to calculate the results by comparing the prediction with the actual value.

4 $$\mathrm{ACC}=\frac{TP+TN}{TP+TN+FP+FN}.$$

When the predicted value is the same as the actual value, the prediction is correct and can be subdivided into TP and TN, as shown in Table 3. There are also two cases of inaccurate predictions: false positive (FP) and false negative (FN). FP refers to when the number of incorrect predictions for a stock price increases by more than 0%, and FN means the number of incorrect predictions of a stock price increases by less than 0%.

**Table 3:** Confusion matrix

	Prediction	
Actual	*TP*	*FN*
	*FP*	*TN*

### Analysis of LSTM Prediction Model Incorporated with Taguchi Method

This study used a combination of chip-based indicators obtained by HGA as the input features for LSTM to evaluate the effect of deep learning on improving prediction accuracy. To solve for the vanishing gradient problem in training the long sequence, it added three control gates to LSTM—input gate, output gate, and forget gate—to determine when to update the memory. The input gate decides which data should enter long-term memory; the output gate identifies which results to output; and the forget gate uses a sigmoid function to establish whether to retain or forget each feature data at a specific time stamp in the cell. Since LSTM generally includes multiple hidden layers, and each hidden layer has multiple neurons, the number of parameters is often significantly large. It is impractical to use the brute force method to find the best parameter combination, given the computational limitations. Thus, this study adopted the Taguchi method to optimize the selection of LSTM parameters.

The Taguchi method uses orthogonal arrays to obtain effective statistical data with fewer experiments. Huang and Tsai [22] used the Taguchi method to determine the optimal combination of factors that may affect the prediction accuracy of SVM. Hsieh et al. [18] used orthogonal arrays to find appropriate hyper-parameters for a backpropagation neural network (BPNN), including the number of neurons in the hidden layer, the learning rate, and the momentum. To avoid the inefficient practice of trial and error, this study utilized orthogonal arrays to optimize LSTM hyper-parameter combinations, including the number of hidden layer neurons, learning rate, batch size, number of epoch, and time steps (Table 4). This study used an L_16_(4^5^) orthogonal array (Table 5). Each factor is set to four levels, and the quality characteristic is prediction accuracy. To avoid interaction effects, the third column of the orthogonal array is intentionally left blank. Each experiment is conducted 3–5 times, and the value for signal-to-noise ratio (S/N) is used to create a response table and diagram to determine the optimal combination.

**Table 4:** Factors and levels of Taguchi orthogonal arrays

Level	Level 1	Level 2	Level 3	Level 4
Factors				
Neuros	16	32	64	128
Batch size	8	16	24	32
Epoch	250	500	750	1000
Time steps	30	45	60	75

**Table 5:** L_16_(4^5^) orthogonal array

Factors	1 (Neuros)	2 (Batch size)	3 (Blank)	4 (Epoch)	5 (Time steps)
Exp					
1	16	8		250	30
2	16	16		500	45
3	16	24		750	60
4	16	32		1000	75
5	32	8		750	75
6	32	16		1000	60
7	32	24		250	45
8	32	32		500	30
9	64	8		1000	45
10	64	16		750	30
11	64	24		500	75
12	64	32		250	60
13	128	8		500	60
14	128	16		250	75
15	128	24		1000	30
16	128	32		750	45

## Analysis and Discussion

### Screen Results for Chip-Based Indicators

This study used GA and three machine learning tools to screen 25 chip-based indicators for the subsequent LSTM analysis. It employed Scikit-Learn, a software machine learning library for Python, to perform machine learning. In addition to the parameters defaulted by the software, the study performed a grid search to identify the best parameter settings for each method (see Table 6).

**Table 6:** Parameter settings for machine learning tools

Machine learning tool	Parameter settings
SVM	C = 495.0, kernel = 'rbf'’, gamma = 1/(number of features + 1)
LR	penalty = 'l2', C = 22.0
DT	criterion = 'gini', splitter = 'best'

To identify the impact of the pandemic on important chip-based indicators affecting stock prices, this study divided the data into two periods: the overall period (from January 1, 2019, to April 29, 2021) and the period after the pandemic (from February 21, 2020, to April 29, 2021). Table 7 presents the HGA classification results for different periods. SVM outperforms the other two methods. Using SVM and LR and post-pandemic data to establish a prediction model can establish better prediction accuracy, whereas DT has no significant effect on prediction accuracy in both periods. Table 7 shows that classifying data under a post-pandemic period can help improve prediction performance.

**Table 7:** Prediction results for HGA using different data periods

	SVM (%)	LR (%)	DT (%)
Overall period	57.22	54.36	57.20
Post-pandemic period	59.65	57.93	56.55

### LSTM Analysis Results Using the Taguchi Method to Adjust Hyper-Parameters

**Fig. 2:**
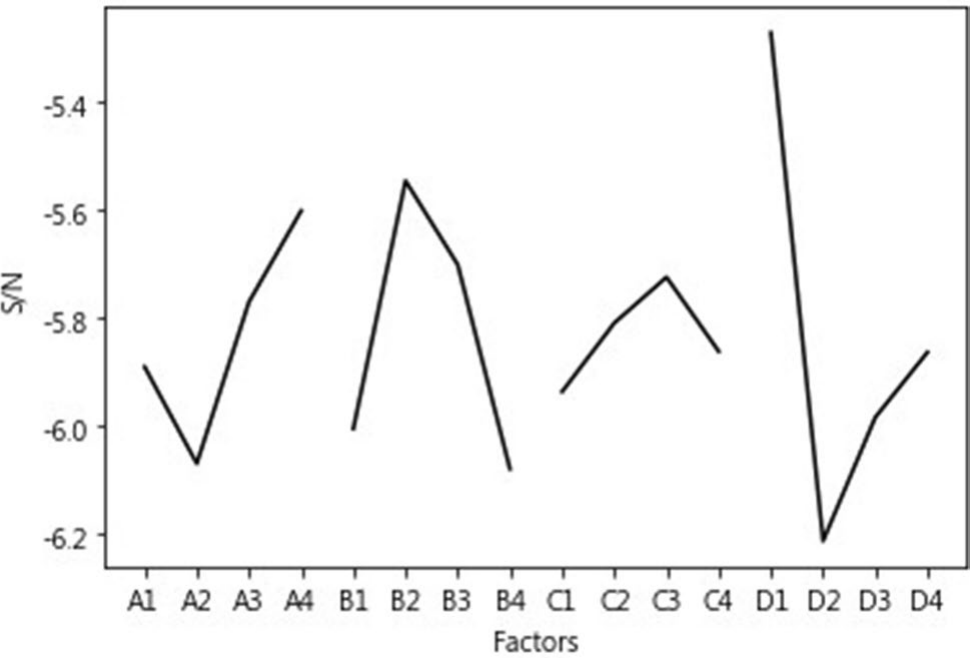
Response diagram for HGA using SVM as the classifier

This study used chip-based indicators screened using HGA as the training features for LSTM. Then, adopting the Taguchi method to determine hyper-parameters, it explored the degree to which LSTM can improve prediction accuracy. After repeated experiments, LSTM was found to more easily converge with five hidden layers. Therefore, this study used five hidden layers to determine the most suitable hyper-parameter combination for modeling. Indicator combinations and sets of optimal hyper-parameters differ by machine learning approach, and thus, Taguchi experiments must be separately conducted. In this study, each experiment with the Taguchi orthogonal array was conducted ten times. The accuracy was averaged to avoid statistical bias. Owing to space limitations, this study only presents the results for HGA using SVM as the classifier in the overall period (Fig. 2).

Among the 16 experiments on the L_16_(4^5^) orthogonal arrays, the best prediction accuracy of 58.89% was reported in the 10th experiment, which was a combination of [A3, B2, C3, D1] (i.e., [64, 16, 750, 30]). It is preferable to have a larger S/N value for the quality characteristic, that is, prediction accuracy. Accordingly, [A4, B2, C3, D1] (i.e., [128, 16, 750, 30]) was selected from Fig. 2 as the most suitable combination of hyper-parameters to build the LSTM models. However, since this combination did not exist in the 16 Taguchi experiments, a confirmation test was required. After analyzing the results of this combination, the prediction accuracy rate reached 60.00%, which was indeed an improvement over the previously estimated highest percentage of 58.89%. Thus, the Taguchi method can effectively optimize LSTM hyper-parameters.

### Quantitative Analysis of Sentiment Variables

Using sentiment variables as a variable to predict stock price fluctuations, this study adopted GRA to evaluate the correlation between the above-mentioned five sentiment variables and stock prices change.

As shown in Table 8, among the five sentiment variables, the negative sentiment score has the highest grey relational grade, followed by the positive sentiment score. Table 9 shows that after the outbreak of the pandemic, the number of discussions about TSMC on PTT increased, and the overall number of articles and total sentiment score were significantly greater than those before the outbreak. Irrespective of the period, the scores for positive and negative sentiments were similar, and the total scores for positive sentiment were slightly higher than those for negative sentiment. The PNS variable ranked third, although simply adding the positive and negative scores of the day does not seem to be an ideal sentiment variable. This is because when a major financial or political event occurs, PTT users tend to speak more enthusiastically, and both positive and negative sentiment scores are high; however, the sum of both scores may be low. This result may be indistinguishable from the case when users express few opinions on the PTT platform. Since NS only represents negative sentiment, and the grey relational grade of PS is close to NS, this study subsequently added both variables to LSTM to model predictions.

**Table 8:** GRA analysis results for five sentiment variables

Sentiment variables	Grey relational grade
PS	0.550
NS	0.572
PNS	0.512
HL	0.460
JS	0.458

**Table 9:** Changes in sentiment score before and after the pandemic

Period	Number of articles	PS score	NS score
Pre-pandemic	485	5732	− 4968
Post-pandemic	1692	16,546	− 14,517
Summation	2177	22,278	− 19,485

### Prediction Model Performance of Sentiment Variables in LSTM

As mentioned in subsection 4.1, using SVM as a classifier in GA can help obtain the best combination of chip-based indicators, including PFL, SITB, SITS, DS, MLS, MLCP, SLB, and SLB. In addition to these eight variables, this study added two sentiment variables, NS and PS, selected using GRA. A total of 10 indicators were included in LSTM to rebuild the prediction model. Table 10 shows that after adding the sentiment indicator, prediction accuracy can reach 62.22%, which is 2.22% higher than the prediction accuracy estimated using the prediction model without sentiment variables. Therefore, adding the two sentiment variables proposed in this study can effectively improve the prediction accuracy of the LSTM model.

**Table 10:** Differences in prediction accuracy after adding sentiment variables

	Prediction accuracy (%)
LSTM without sentiment variables	60.00
LSTM with sentiment variables	62.22
Improvement	+ 2.22

### Prediction Performance Evaluation

Several studies have discussed stock market forecasting, and each uses different forecasting variables and tools. However, unlike the research on image recognition that generally uses the same database as the basis for comparison, studies on the accuracy of stock market forecasts do not have the same database as a benchmark for comparison. This is because research objects and time periods differ by study. Thus, it is difficult to objectively compare forecast performance with those presented in other papers. In addition to demonstrating the improvement of prediction accuracy by applying LSTM and the sentiment of social media, this study also employs different machine learning tools, including DT, LR, and SVM, in the GA-based feature selection process to compare the performance of different classifiers. Moreover, we focus on the comparison with that of Huang and Liu [21] since their work is the most analogous to our study.

Huang and Liu [21] also include chips-based indices and the sentiment of social media in the prediction model. Their article uses HHPIC as the analysis object, and this study focuses on TSMC. The analysis period (110 days) in their article does not account for the COVID outbreak, whereas our study covers the period before and after the outbreak (from January 1, 2019 to April 29, 2021, 561 days). While their article also considers the sentiment score as a predictive variable, they only use a single sentiment variable. Furthermore, they use chips-based indices as predictive variables but employ LR only as a predictive and feature screening tool.

Although, Huang and Liu’s prediction accuracy appears marginally higher than that in this study, the higher prediction accuracy can be attributed to the lack of analyzed data. Their study uses a small number of days for the analysis. For example, their study shows *P* = 1% (stock price increased more than 1%) and a prediction accuracy of as high as 78%. During the analysis period, HHPIC showed an increase of more than 1% for only 40 days, which is significantly less than the number of days it reported *P* = 0% (110 days). Thus, it is difficult to use LR to tackle the issue of imbalanced data, which may easily occur when analyzing data for 40 days. Thus, while the accuracy of their analysis is impressive, there remain several concerns. According to Westreich et al. [48], if the number of observations is less than the number of features, using LR may lead to overfitting. Therefore, as stated in Sect. 2 of our paper, Huang and Liu’s analysis results are not statistically significant owing to the relatively small dataset.

## Conclusions

This study makes four major contributions. First, it integrated GA with three machine learning tools to identify the best combination of indicators, which were then incorporated into LSTM to improve prediction accuracy. Second, after the pandemic, the social media sentiment score significantly increased, and the composition of the best chip-based indicators for the LSTM prediction model changed. This study confirmed that segregating data between periods before and after the pandemic can help improve prediction performance. Third, using Taguchi’s method to find the best combination of LSTM hyper-parameters can efficiently enhance LSTM prediction performance. Finally, using GRA, this study identified that sentiment variables NS and PS are most strongly correlated with fluctuations of TSMC stocks, and adding the two sentiment variables to the LSTM model can improve the prediction performance for stock price fluctuation.

This study is subject to certain limitations that warrant further consideration. First, it only used articles on PTT as a source of information. However, investors do not solely rely on PTT as an information source. Future research may explore other social media platforms to ensure information diversity. Second, internet languages or slang rapidly change, and many users use grammar ironically or sarcastically. Thus, it may be inaccurate to judge emotions using the current phrase rule method. To more efficiently judge the sentiment of online messages, artificial intelligence methods can be used in future analyses. Third, this research only made binary predictions on the rise or fall of stock prices on the next day. Further research is needed on formulating actual trading strategies on the basis of analysis results.

## Data Availability

The datasets generated during and/or analyzed during the current study are available from the corresponding author on reasonable request.

## Abbreviations

ACC: Prediction accuracy
ANN: Artificial neural network
ANSI: Aggregate news sentiment index
ARIMA: Autoregressive integrated moving average model
ATNN: Adaptive time delay neural networks
CNN: Convolution neural networks
COVID-19: Coronavirus disease 2019
DB: Dealer buy
DBN: Deep belief networks
DJIA: Dow Jones Industrial Average
DNN: Deep neural networks
DOMPSL: Day offset of margin purchasing and short selling
DS: Dealer sell
DTN: Day trading number
DTT: Day trading turnover
DTV: Day trading volume
FCIB: Foreign/Chinese investors buy
FCIS: Foreign/Chinese investors sell
FN: False negative
FP: False positive
GA: Genetic algorithm
GRA: Grey relational analysis
HGA: Hybrid genetic algorithm
HHPIC: Taiwan Hon-Hai Precision Industry Co., Ltd.
HL: Huang and Liu sentiment score
ISSI: Islamic Sharia stock index
JS: Jing sentiment score
KNN: K-nearest neighbor
KOSPI: Korean stock price index
LR: Logistic regression
LSTM: Long short-term memory
MBL: Margin balance long
MBS: Margin balance short
MLB: Margin loan buy
MLCP: Margin loan cash pay
MLS: Margin loan sell
NBSTII: Net buy/sell of three institutional investors
NS: Negative sentiment score
NST: Number of shares traded
PNS: Positive and negative score
PS: Positive sentiment score
PTT: PTT bulletin board system
PFL: Price fluctuation limit
RAF: Random forests
RNN: Recurrent neural networks
RSI: Relative strength index
RWT: Random walk theory
SITB: Securities investment trust buy
SITS: Securities investment trust sell
SLB: Stock loan buy
SLCP: Stock loan cash pay
SLS: Stock loan sell
SSE: Shanghai stock exchange
SVM: Support vector machine
TIV: Turnover in value
TN: True negative
TP: True positive
TSNN: Time delay neural networks
TSMC: Taiwan Semiconductor Manufacturing Company
TV: Trading volume
TVIX: Taiwan volatility index
